# An Automatic Method for Nucleus Boundary Segmentation Based on a Closed Cubic Spline

**DOI:** 10.3389/fninf.2016.00021

**Published:** 2016-06-16

**Authors:** Zhao Feng, Anan Li, Hui Gong, Qingming Luo

**Affiliations:** ^1^Britton Chance Center for Biomedical Photonics, Wuhan National Laboratory for Optoelectronics- Huazhong University of Science and TechnologyWuhan, China; ^2^Key Laboratory of Biomedical Photonics of Ministry of Education, College of Life Science and Technology, Huazhong University of Science and TechnologyWuhan, China

**Keywords:** closed cubic spline, brain, histological image, nucleus segmentation, automatic

## Abstract

The recognition of brain nuclei is the basis for localizing brain functions. Traditional histological research, represented by atlas illustration, achieves the goal of nucleus boundary recognition by manual delineation, but it has become increasingly difficult to extend this handmade method to delineating brain regions and nuclei from large datasets acquired by the recently developed single-cell-resolution imaging techniques for the whole brain. Here, we propose a method based on a closed cubic spline (CCS), which can automatically segment the boundaries of nuclei that differ to a relatively high degree in cell density from the surrounding areas and has been validated on model images and Nissl-stained microimages of mouse brain. It may even be extended to the segmentation of target outlines on MRI or CT images. The proposed method for the automatic extraction of nucleus boundaries would greatly accelerate the illustration of high-resolution brain atlases.

## Introduction

The brain consists of massive nuclei with different functions. In neuroscience research, the precise recognition and delineation of nucleus boundaries is the crux of brain area identification and atlas illustration.

The contemporary automatic recognition methods for nucleus boundaries are mainly based on MR images (Wiegell et al., 2003; van der Lijn et al., 2008) and micro-optical images (McDonald, 1982; Geisler et al., 2003). In MR images and low-resolution histological images, the distribution of gray level in a nucleus is homogenous, facilitating the application of many classical image-segmentation techniques (Balafar et al., 2010). In contrast, nuclei on cytoarchitectural images at single-cell resolution do not exhibit an even and continuous distribution of the gray level but, rather, clusters of discrete recognizable neurons. Microscopic images clearly show that the cell morphologies and cell densities of brain nuclei differ from each other, resulting in blurry borders between nuclei and their neighboring areas (Gahr, 1997). The illustration of traditional histological brain atlases, for instance, Brodmann's human brain atlas (Brodmann, 1908), the mouse brain in stereotaxic coordinates (Paxinos and Franklin, 2004) and the Allen Reference Atlas (Dong, 2008), relies on the experience of anatomical experts (Brunjes et al., 2005), who draw the boundaries of nuclei with smooth curves on a limited number of sections according to the morphology and density differences of the imaged cells. These handmade atlases have been widely used in neurobiological research. However, given the rise of whole-brain continuous imaging techniques with single-cell resolution (Li et al., 2010), the data volume of sections from a single mouse brain has exceeded what was acquired with traditional methods. As a result, it has become unrealistic to manually segment the boundaries of brain regions and nuclei. Thus, the development of method for the automatic recognition and segmentation of entire and continuous boundaries of nuclei on large, high-resolution histological images is urgently needed.

Some efforts have been made for automatic parcellation of brain regions and nuclei on single-cell-resolution histological images. Xiang et al. segmented the abducens nucleus based on the Gabor wavelet, which yielded discontinuous boundaries (Xiang et al., 2004). Mesejo et al. segmented the pyramidal cell layer and granular cell layer of the hippocampus formation (Mesejo et al., 2012, 2013). However, their selected sections were thick, and the cells of the two segmented structures overlapped one another, resulting in a homogeneous distribution of the gray level. The Otsu thresholding and Chan-Vese model applied in their work were only suitable for the segmentation of these homogeneous regions, not targets made up of isolated cells. Amunts et al. automatically acquired the dividing lines between neighboring brain regions in the cortex by extracting the curve features from gray level index profiles on Nissl-stained images (Amunts et al., 2013). Meyer et al. divided the anterior olfactory nucleus region into several parts by statistically analyzing the cell distribution (Meyer et al., 2006). However, they only manually delineated the outline of brain structures and then automatically computed the dividing lines among the subregions within; they did not directly segment an entire closed outline of the brain regions and nuclei from original cytoarchitectural images. Briefly, the methods mentioned above all have some shortcomings in segmenting continuous boundaries of nuclei consisting of isolated cell bodies.

In this paper, we propose a method to automatically segment the outlines of nuclei whose cell densities differ to a relatively high degree relative to their surrounding areas. This method uses a closed cubic spline (CCS) as its action object and guides the automatic evolution of CCS in the whole image sequence based on the features extracted from the surrounding images of the CCS control points, and the entire closed outline of the target nucleus on the whole image sequence was successfully acquired. The proposed method was applied to nuclei with densely packed cells, such as the mitral cell layer of the olfactory bulb, and nuclei with low cell density but surrounded by areas of high cell density, such as the molecular layer of the cerebellar lobe. This method was also extended to the segmentation of non-nucleus targets.

## Method

**Figure 1:**
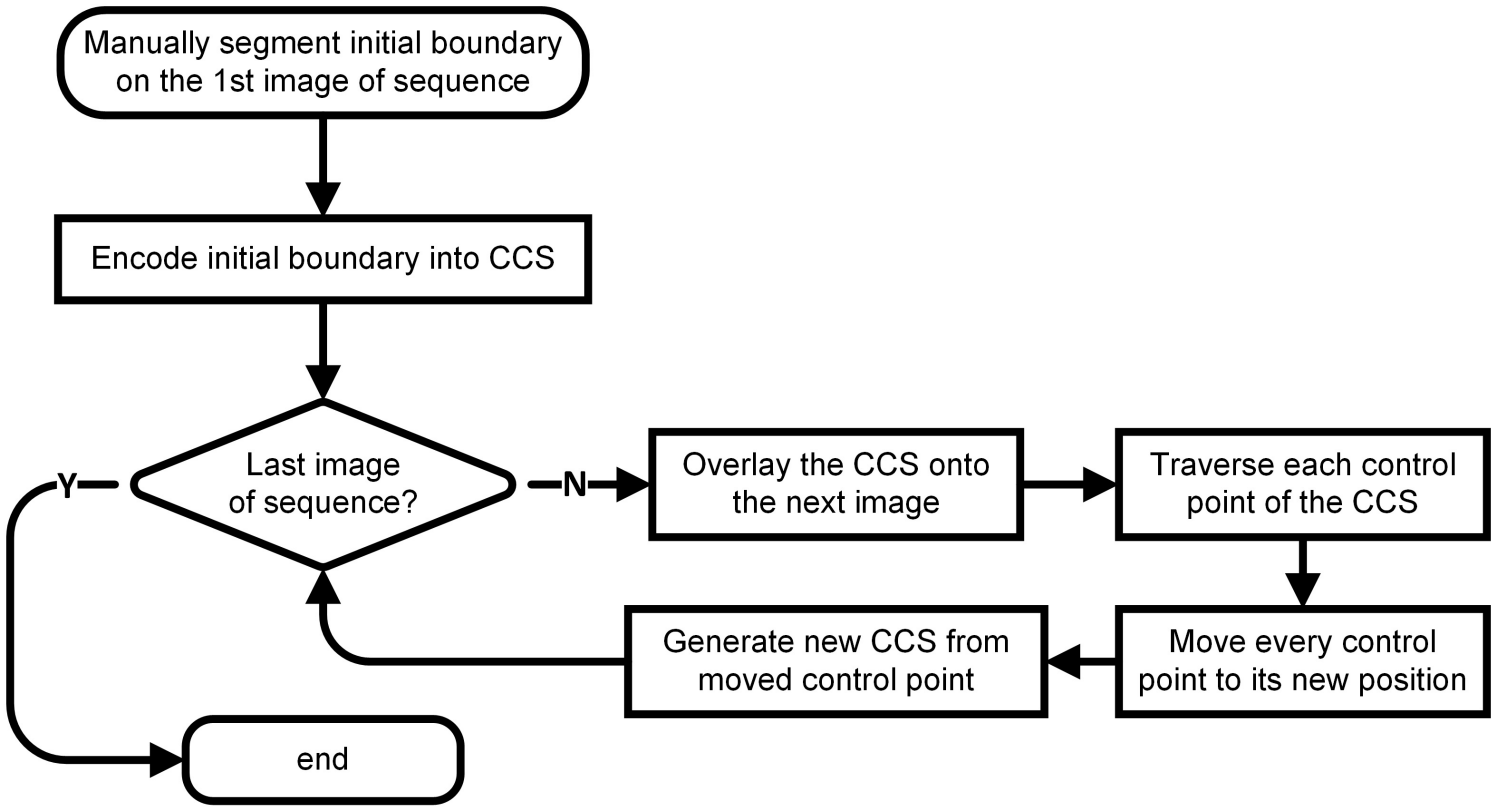
**The workflow**.

The whole workflow is shown as Figure 1. First, we manually delineated the boundary of the target nucleus on the first image of the given image sequence and extracted the feature points of the boundary, which we used to construct a CCS that was overlaid on the image next to the first one. Then, we traversed each control point of the CCS and moved each to its new position on the new image by calculating the image similarities and maximizing the difference between the inner and outer sides of each control point. After traversing all control points, we generated a new boundary based on the control points at their new positions. We repeated this process throughout the image sequence. A detailed explanation of every step of our method follows.

### Manual segmentation of the initial boundary and extraction of control points

The manual segmentation of the boundary of nucleus on the first image of the sequence was implemented by the Segmentation Editor module of Amira (FEI, Hillsboro, OR, USA). The segmented boundary was saved as a label image, in which the gray value of the pixels inside the boundary was 255 and that of the ones outside the boundary was 0.

Using the corner detection algorithms of Harris (Harris and Stephens, 1988), Log (Lindeberg, 1998) and Gilles (Gilles, 1999), we extracted the points that could describe the shape features of the segmented boundary, which could then be used as CSS control points.

### CCS encoding of the initial boundary

The CCS was a type of smooth and closed curve comprising n control points and n segments. At each control point, the CCS was continuous and differentiable (Press, 2007). The CCS was deduced from the control point set according to Kershaw (1972).

The coordinates of any point on the *i*th segment of the CCS could be interpolated by the following equation set depending on variable *t* (*t*∈[0, 1]):

(1) $$\begin{array}{rcl} x\left(t\right) & = & b_{x1}^{i}+b_{x2}^{i}t+b_{x3}^{i}t^{2}+b_{x4}^{i}t^{3} \end{array}$$

(2) $$\begin{array}{rcl} y\left(t\right) & = & b_{y1}^{i}+b_{y2}^{i}t+b_{y3}^{i}t^{2}+b_{y4}^{i}t^{3} \end{array}$$

where *b*_*xj*_ and *b*_*yj*_ (*j* = 1, 2, 3) are the parameters of the *i*th segment of the CCS. When variable *t* was given a value 0 or 1, the values of *x* and *y* corresponded to the coordinates of the start or end point of the *i*th segment, respectively. Eight parameters were needed to describe one segment, and thus, a total of 8n parameters is required to describe the whole CCS. Note that the expressions of *x*(*t*) and *y*(*t*) are symmetrical. We deduced *b*_*x*1_−*b*_*x*4_ in Equation (1) as an example, which can be extended to the deduction of a total of 4*n* parameters of the *x* coordinate of CCS.

The CCS was denoted as *C*, and the start and end points of the *i*th segment of *C* were marked as *P*_*i*−1_ and *P*_*i*_, respectively. We denoted the control points of *C* to be *P* = {*P*_1_, *P*_2_, …, *P*_*n*_}, and thus, for the *i*th segment of *C*, we have

(3) $$\begin{array}{l} \{\begin{array}{l} x(0)=b_{x1}^{i}=P_{i-1}(x)\\ x(1)=b_{x1}^{i}+b_{x2}^{i}+b_{x3}^{i}+b_{x4}^{i}=P_{i}(x)\\ \frac{dx(0)}{dt}=b_{x2}^{i}={P^{\prime }}_{i-1}(x)\\ \frac{dx(1)}{dt}=b_{x2}^{i}+2b_{x3}^{i}+3b_{x4}^{i}={P^{\prime }}_{i}(x) \end{array} \end{array}$$

where *P*_*i*−1_(*x*) and *P*_*i*_(*x*), respectively, are the *x* coordinates of *P*_*i*−1_ and P_i_, and ${P^{\prime }}_{i-1}\left(x\right)$ and ${P^{\prime }}_{i}\left(x\right)$ are the first derivatives of the *x* coordinates at *P*_*i*−1_(*x*) and *P*_*i*_(*x*) with respect to *t*. According to (3), we have

(4) $$\begin{array}{l} \{\begin{array}{l} b_{x1}^{i}=P_{i-1}(x)\\ b_{x2}^{i}={P^{\prime }}_{i-1}(x)\\ b_{x3}^{i}=3P_{i}(x)-{P^{\prime }}_{i}(x)-2{P^{\prime }}_{i-1}(x)-3P_{i-1}(x)\\ b_{x4}^{i}={P^{\prime }}_{i}(x)+{P^{\prime }}_{i-1}(x)+2P_{i-1}(x)-2P_{i}(x) \end{array} \end{array}$$

Given the continuous and differentiable characteristics of the CCS at any control point, we have

(5) $$\begin{array}{l} \frac{d^{2}P^{i}\left(x-0\right)}{dt^{2}}=\frac{d^{2}P^{i}\left(x+0\right)}{dt^{2}} \end{array}$$

Substituting the second-derivative expression of the CCS into Equation (5), we have

(6) $$\begin{array}{l} 2b_{x3}^{i}+6b_{x4}^{i}=2b_{x3}^{i+1} \end{array}$$

Then, combining Equations (4) and (6) to eliminate parameter *b*, we can acquire the equation set with respect to *P* and *P*′:

(7) $$\begin{array}{l} \{\begin{array}{l} 4{P^{\prime }}_{1}(x)+{P^{\prime }}_{2}(x)+{P^{\prime }}_{n}(x)=3P_{2}(x)-3P_{n}(x)\\ \begin{array}{l} {P^{\prime }}_{i-1}(x)+4{P^{\prime }}_{i}(x)+{P^{\prime }}_{i+1}(x)=3P_{i+1}(x)-3P_{i-1}(x),i\\ \;=2,3,\ldots ,n-1 \end{array}\\ {P^{\prime }}_{1}(x)+{P^{\prime }}_{n-1}(x)+4{P^{\prime }}_{n}(x)=3P_{1}(x)-3P_{n-1}(x) \end{array} \end{array}$$

The above equations can then be rewritten as a matrix multiplication expression, as shown below:

(8) $$\begin{array}{l} \left[\begin{array}{cccccc} 4 & 1 & 0 & \ldots & 0 & 1\\ 1 & 4 & 0\\ 0 & \ldots & \ldots \\ \ldots & \ldots & 0\\ 0 & 4 & 1\\ 1 & 0 & \ldots & 0 & 1 & 4 \end{array}\right]\left[\begin{array}{c} P_{1}^{\prime }\\ P_{2}^{\prime }\\ \ldots \\ \ldots \\ P_{n-1}^{\prime }\\ P_{n}^{\prime } \end{array}\right]=3\times \left[\begin{array}{c} P_{2}\\ P_{3}\\ \ldots \\ \ldots \\ P_{n}\\ P_{1} \end{array}\right]-3\times \left[\begin{array}{c} P_{n}\\ P_{1}\\ \ldots \\ \ldots \\ P_{n-2}\\ P_{n-1} \end{array}\right] \end{array}$$

By solving the matrix Equation (8), we obtain ${P^{\prime }}_{i}\left(x\right)$, where i = 1,2,…,*n*. After substituting the values of ${P^{\prime }}_{i}\left(x\right)$ into Equation (4), we attain the 4*n* parameters for the *x* coordinate of the CCS. The same procedure was used to acquire the 4*n* parameters for the *y* coordinate.

### Control point evolution of CCS

**Figure 2:**
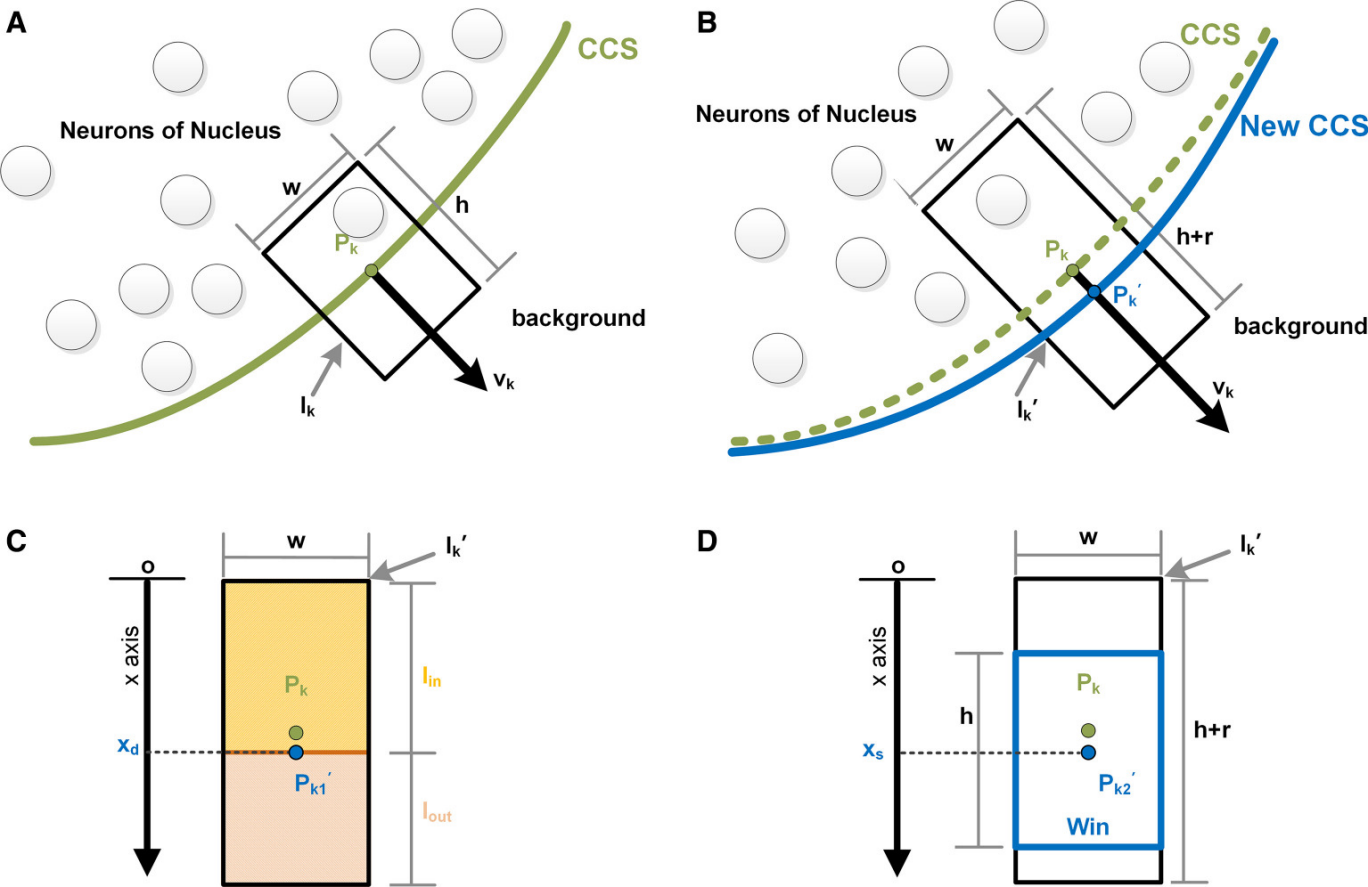
**Control point evolution**. **(A)** The *n*th image of the sequence. The green curve is a segment of the computed CCS in the current image. *P*_*k*_ is the *k*th control point of CCS, and *v*_*k*_ is the normal vector at the point *P*_*k*_. Centered on *P*_*k*_, the black rectangle *I*_*k*_ is the surrounding image of *P*_*k*_, which was extracted along *v*_*k*_, with width *w* and height *h*. **(B)** The *n+1*st image of the sequence. The green curve is the CCS segment of the *n*th image, and the blue curve is the evolved new CCS segment, on which the control point *P*_*k*_moved to its new position along *v*_*k*_ and is denoted $P_{k}^{\prime }$. The black rectangle $I_{k}^{\prime }$ is the surrounding image of *P*_*k*_, with width *w* and the height *h*+*r*. **(C)** $I_{k}^{\prime }$ is divided by the moving point into two parts: *I*_*in*_ and *I*_*out*_. When the difference in the mean gray values of *I*_*in*_ and *I*_*out*_ is maximized, the moving point is labeled $P_{k1}^{\prime }$, and its coordinate is *x*_*d*_. **(D)** Centered on the moving point, a sliding window Win is constructed. When the content of Win reaches maximal similarity with *I*_*k*_, the moving point is marked as $P_{k2}^{\prime }$, and its coordinate is *x*_*s*_.

To iterate each control point of the CCS, at control point *P*_*k*_, we extracted the surrounding image *I*_*k*_along the normal direction *v*_*k*_ of *P*_*k*_. The width and height were *w* and *h*, respectively, as shown in Figure 2A. We then overlaid the CCS onto the next section of the image sequence and extracted the surrounding image $I_{k}^{\prime }$ of *P*_*k*_ along its normal direction, as shown in Figure 2B. The width of $I_{k}^{\prime }$ was also *w*, whereas the height was the height of *I*_*k*_ with a sliding range *r*. We let the point *P*_*k*_ slide along *v*_*k*_ in $I_{k}^{\prime }$ and denoted the moving *P*_*k*_ as $P_{k}^{\prime }$. We defined the distance of $P_{k}^{\prime }$ and the bottom edge of $I_{k}^{\prime }$ as *x*. A sliding window *Win*, whose height was *h* and width was *w*, was then constructed along the direction of *v*_*k*_ with $P_{k}^{\prime }$ as its center. The value of *x* was required to be an integer with pixel as the unit, and thus, there were only *r* possible values of *x*.

As shown in Figure 2C, with a line through $P_{k}^{\prime }$, $I_{k}^{\prime }$ can be divided into an inner part and outer part, which are denoted as *I*_*in*_and *I*_*out*_, respectively. The mean gray values of these two parts are indicated as *mean*_*in*_ and *mean*_*out*_, respectively. When *P*_*k*_ moved to the position marked $P_{k1}^{\prime }$ where |*mean*_*in*_−*mean*_*out*_| was maximized, the value of *x* was denoted *x*_*d*_, indicating that the difference between *I*_*in*_ and *I*_*out*_ was maximized. Then, we calculated the similarities between *Win* and *I*_*k*_ given all possible values of *x*. The measurements of similarity included the sum the variance of the gray value, the covariance, the correlation coefficient and the angle cosine. The values of *x* with maximal similarity under the 4 criteria were recorded and sorted. The median of the 4 values of *x* is denoted *x*_*s*_ and was regarded as the value of *x* at which *W*_*in*_ and *I*_*k*_ exhibited the best match, as shown in Figure 2D.

The value of *x* was determined by both *x*_*d*_ and *x*_*s*_. We constructed a minimum object function as indicated below, where α and β were constant:

$$\begin{array}{l} f=\alpha {\left(x-x_{s}\right)}^{2}+\beta {\left(x-x_{d}\right)}^{2} \end{array}$$

The value of *x* that minimized the object function *f* was

$$\begin{array}{l} x=\frac{\alpha }{\alpha +\beta }\;x_{s}+\frac{\beta }{\alpha +\beta }\;x_{d} \end{array}$$

Therefore, *x* can be seen as a linear combination of *x*_*d*_ and *x*_*s*_. As α increased, *x*_*s*_ weighed more, indicating that the value of *x* was increasingly related to the similarity of the two images surrounding the control point on neighboring sections; as β increased, *x*_*d*_ weighed more, and thus, *x* was more determined by the difference between the inner and outer parts of the surrounding image divided by the control point. According to our experience, the values of α and β were both set to 0.5.

Subsequently, the point *P*_*k*_ moved to its new position, as shown in Figure 2B. The calculation above was performed for every control point of the CCS, giving the new positions of all the control points.

### Reconstruction of CCS from evolved control points

The method of constructing a new CCS from control points with new coordinates was the same as described above. Note that if the shape of a contour was complicated, the neighboring nodes of the CCS might exchange their positions, causing intersections of the CCS around these control points. To avoid these intersections, we applied the procedures below:

1. Calculate the distance between every control point and its *n*th (*n* = 1, 2, …, *m*−1, where *m* is the number of control points of CCS) neighboring control points on the right side;
2. Define *D* as the index depth, *T*1 as the thresholding of the nearest distance between control points, and any control point together with any of its right-side neighbor as a control point pair. Find all of the control point pairs with distances less than *T*1 and differences between their index numbers less than *D*. If the distance between the two points of a control point pair was less than *T*1, remove one from the pair and the points in between.
3. Calculate the distance between the remaining control points and insert new control points between the neighboring control points with distances exceeding a manually assigned maximal distance threshold *T*2.

The values of *T*1, *T*2 and *D* were set to 10, 20 and 4, respectively, based on our experience.

### Design of CCS toolkit

We also developed a Matlab toolkit named CCSToolkit, which can be downloaded from https://github.com/VBNProject/CCS-Segmentation-Toolkit. This toolkit includes 22 functions that implement the code necessary for our method and the common operations on the CCS. The functions in the toolkit can be grouped into 3 categories: construction/decomposition of the CCS, information extraction and CCS computation.

### Construction method of model data

To validate our method, we created several image sequences that simulated nuclei formed by packed cells with randomly generated particles. The process was as follows.

1. In a limited space (200 × 200 × 200 voxels), we defined a spherical area with radius 60 and center coordinate (100, 100, 100) as the target nucleus. The remaining area was defined as background.
2. We traversed every voxel in the created 3D space. For voxels in the nucleus and background areas, we generated randomly distributed particles with radii in the range [2, 3] and gray level values in the range [140, 255] by different probabilities *P*_*o*_ and *P*_*b*_, respectively, to simulate different cell densities within and outside the target nucleus.
3. We stored the created 3D space as an image sequence.

### Evaluation method

We evaluated the results obtained using our method on model images using a supervised evaluation strategy. The chosen evaluation parameters were the Dice coefficient (DC) (Dice, 1945) and the Hausdorff distance (Huttenlocher et al., 1993). NHD is used for measuring the similarities between the segmentation results and real boundaries, while DC is used for calculating the overlapping of segmented area and the area surrounded by real boundaries.

In any given image from the model image sequence, we defined the real area occupied by the target nucleus as *A*_*r*_, and the calculated area as *A*_*c*_; thus, the DC was defined as follows:

$$\begin{array}{l} \kappa =\frac{2|A_{r}⋂A_{c}|}{\left|A_{r}|+|A_{c}\right|} \end{array}$$

Here, for convenience, the area was approximated as the pixel number, and *A*_*r*_ was defined as the number of pixels occupied by the nucleus area. Considering that the randomly generated particles belonging to the nucleus area could be on the boundary of the nucleus, we adopted the convention that the outside area of these particles was included in *A*_*r*_.

We then defined *S*_*r*_ to be the real boundary of the nucleus area and *s*_*r*_ to be any pixel that belonged to *S*_*r*_. Thus, the shortest distance from any pixel *p* to *S*_*r*_ was

$$\begin{array}{l} d\left(p,S_{r}\right)=\underset{s_{r}\in S_{r}}{\min}||p-s_{r}|| \end{array}$$

The Hausdorff distance was defined as

$$\begin{array}{l} HD\left(S_{r},S_{c}\right)=\max\left\{\underset{s_{r}\in S_{r}}{\max}d\left(s_{r},S_{c}\right),\underset{s_{c}\in S_{c}}{\max}d\left(s_{c},S_{r}\right)\right\} \end{array}$$

where *S*_*c*_ was defined as the calculated boundary of the nucleus and *s*_*c*_ as any pixel that belonged to *S*_*c*_.

To avoid the influence of the target's area on the value of the Hausdorff distance, we normalized the Hausdorff distance by the real perimeter of the target area as below:

$$\begin{array}{l} NHD\left(S_{r},S_{c}\right)\;=\frac{HD\;(S_{r},S_{c})}{S_{r}} \end{array}$$

## Results

We validated our method using the 4 created models and applied the DC and Normalized Hausdorff distance (NHD) to evaluate the precision of our method. Next, we tested our method on Nissl-stained mouse brain images acquired by Micro-Optical Sectioning Tomography system (MOST) (Li et al., 2010). Finally, we applied our method to the segmentation of the outline of a mouse brain using the coronal sections acquired by MRI and CT.

### Model testing

**Figure 3:**
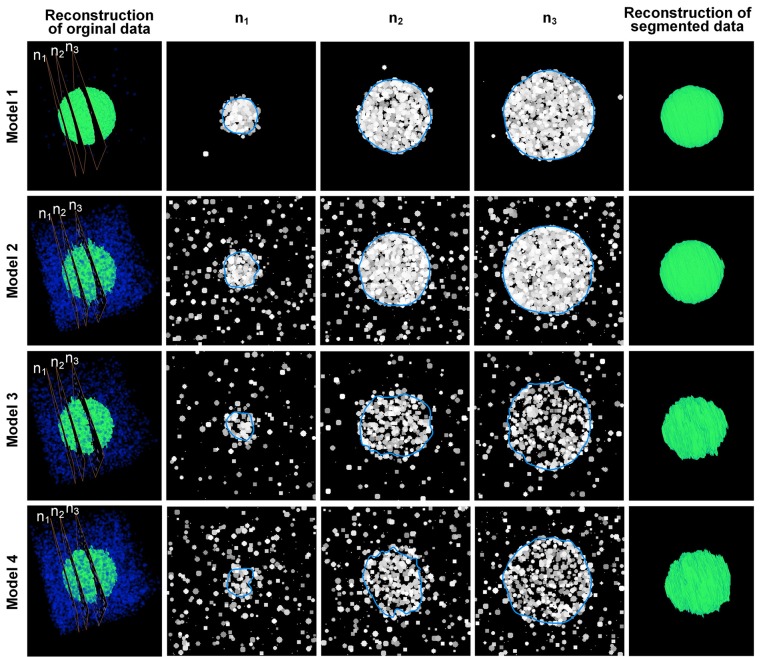
**Results of the model data**. The 1st–4th rows show the computational results obtained using 4 model datasets. The 1st column is the 3D reconstruction of the image sequence of the model data. Blue indicates the “cells” in the background area, whereas green indicates the “cells” in the nucleus area. n1 to n3 are 3 sections chosen from the image sequences that correspond to the 2nd–4th columns. The blue curves in the 2nd–4th columns are the computed CCS of the outline of the “nuclei” in the model data. The 5th row shows the 3D reconstruction of the computation results. The (*P*_*o*_, *P*_*b*_) parameters of the 4 models are (2%, 0.001%), (2%, 0.1%), (0.7%, 0.05%), and (0.7%, 0.1%), respectively.

Using different *P*_*o*_ and *P*_*b*_ values, we created 4 sets of model image data, which are shown in the 1st-4th rows in Figure 3. In model 1, the probability of the occurrence of particles in the target area was much higher than in the background area; thus, the particles in the target area were densely packed, and the boundary of the target area was the most unambiguous of all the tested models. In model 2, the probability of the occurrence of particles in the background area was higher than that in model 1, which could disturb the decision making about the boundary of the target area. In model 3, the possibility of particles occurring in the target area was lower than that in model 1, resulting in a blurry boundary around the target area. In model 4, the possibility of the occurrence of particles in the background area was higher than that in model 3, and this model had the blurriest boundaries.

The values of the parameters *w*, *h* and *r* of model 1 and model 2 were set to 30, 20, and 20, respectively. For model 3 and model 4, the aforementioned parameters were set to 80, 40, and 20, respectively, to reduce the effects of the particles of the background area. Figure 3 shows that our method could extract the boundaries of nuclei in areas with different nucleus-area densities and background densities. It could also be seen that some particles next to the computed boundaries were not included. These particles were firstly appeared isolatedly around the target nucleus. When these particles were starting to contact with the border of nucleus, our method would not include them into the segmented boundary immediately, until more particles appeared around these particles in the next section of the image sequence. This strategy can reduce the influence of noises effectively.

**Figure 4:**
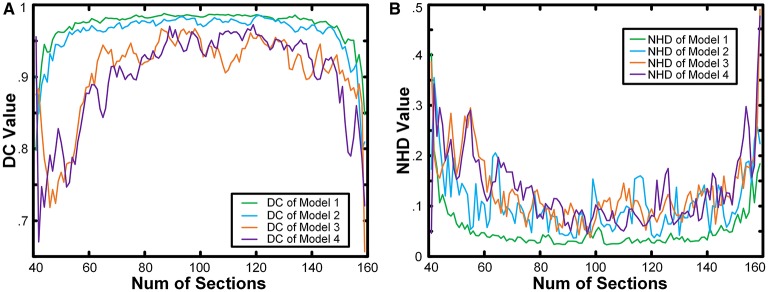
**Quantitative analysis of the results obtained from model images**. The NHD and DC of the results obtained from the 4 model image datasets. The horizontal axis is the number of images in the sequence. The vertical axis in **(A)** is the NHD value, whereas in **(B)**, it is the DC value.

We calculated the boundaries of the nucleus from the 41st to the 159th images of the whole model dataset and compared the results with the real boundaries according to NHD and DC. The statistical results are shown in Figure 4. In the first and last images of the sequence, in which the target nucleus appeared and vanished, because the area was small, the segmentation result could have been more easily influenced by particles outside the nucleus, leading to a higher NHD value and a lower DC value and implying a higher bias of the calculation results obtained using the real boundaries. However, the DC values of all the images in the 4 models exceeded 0.65, and the NHD values were lower than 0.5. In different models, as the content of the model image became more complicated and the particle density of the nucleus area decreased, the DC value decreased gradually, and the NHD value increased slowly, which corresponded to a slow decrease in the segmentation precision. The mean value and standard deviation of the NHD and DC values are listed in the first and second column of Table 1. From the table, we can see that the average DC of each model image exceeded 0.90 and that the NHD was lower than 0.15. We also evaluated the 3D reconstruction results of 4 models with DC. The results are also shown in the third column of Table 1. We could see that all the DC values of the 4 models are above 0.90, which shows a good match between our segmentation results and the real boundaries.

**Table 1:** **Statistical analysis of the results obtained from model images**.

	**NHD**	**DC**	**DC (3D)**
Model 1	0.052 ± 0.047	0.972 ± 0.027	0.981
Model 2	0.105 ± 0.057	0.961 ± 0.032	0.972
Model 3	0.130 ± 0.068	0.907 ± 0.061	0.927
Model 4	0.133 ± 0.073	0.900 ± 0.067	0.926

### Test on real cytoarchitectural images

To validate our method for single-cell-resolution image sequences, we selected a 3D mouse whole-brain dataset with 0.35-μm horizontal resolution and 1-μm axial resolution acquired by the MOST system and saved as consecutive coronal sections. The dataset was acquired from an 8-week-old C57BL/6 male mouse (Jackson Laboratory). All of the animal experiments followed procedures approved by the Institutional Animal Ethics Committee of Huazhong University of Science and Technology.

**Figure 5:**
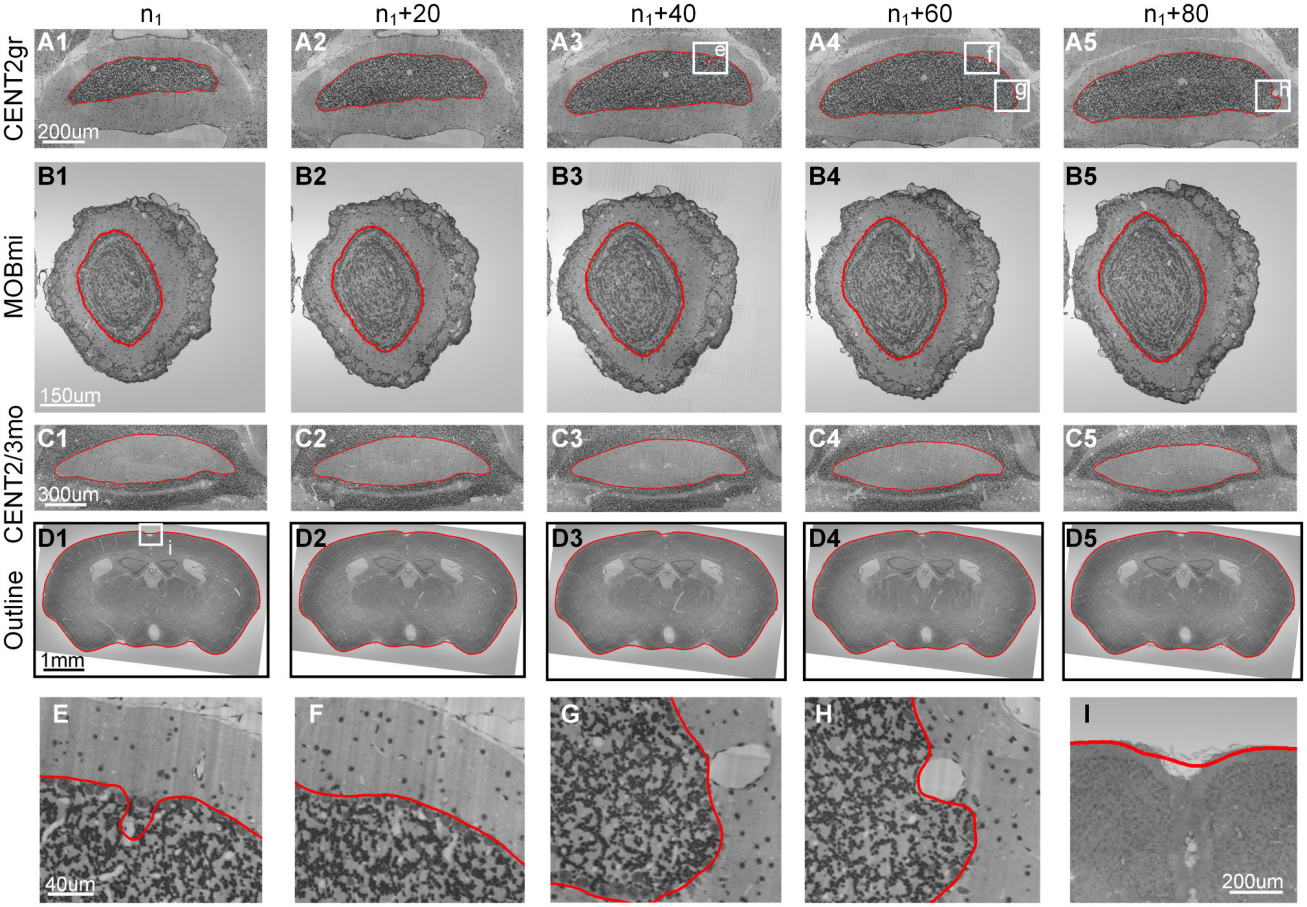
**Results from cytoarchitectural images**. The results obtained by applying our method to 4 cytoarchitectural image datasets. **(A1–D5)** The 1st–4th rows present the contour evolution processes of CENT2gr, CENT2/3mo, MOBmi and the whole mouse brain. **(E–I)** The 5th row gives the computational details. The red curves are the computed CCS. n_1_ represents the 20th section of the image sequence of every dataset. The image to the right of each column is the 20th image after it.

We selected the image sequence containing the granular layer of Lobule II (CENT2gr) to demonstrate the performance of our method in a typically encountered situation. The cells in CENT2gr are compact, whereas the cells in the surrounding area are sparse, which was similar to model 3. Based on our method, we correctly extracted the boundaries of this selected nucleus in 100 consecutive coronal sections (Figures 5A1–A5).

We then used the mitral layer of the main olfactory bulb (MOBmi) to evaluate our method in a more complicated situation. The MOBmi is a very thin circular area with relatively high cell density. The outside and inside of the MOBmi are the outer plexiform layer (MOBopl) and inner plexiform layer (MOBipl), respectively. The cell densities of these two nuclei are lower than those of MOBmi but not drastically so. We evaluated our method on a series of 100 coronal sections to determine the border between MOBmi and MOBopl, as shown in Figures 5B1–B5.

Our method can also be applied to extract the boundaries of nuclei with low cell density that are surrounded by areas with high cell density. On coronal sections of mouse brain, the molecular layer of Lobule II (CENT2mo) and Lobule III (CENT3mo) of the cerebellum have low cell density and are surrounded by the granular layers of Lobule II (CENT2gr) and Lobule III (CENT3gr). Using our method, we extracted the boundary of CENT2mo and CENT3mo as a whole (Figures 5C1–C5).

Finally, this method could even be applied to the segmentation of traditional non-nucleus objects with an even distribution of gray level values. We used the outline of the mouse brain in coronal sections for demonstration. We manually segmented the outline of the mouse brain in the first section and then calculated the outline of the mouse brain in the subsequent sections (Figures 5D1–D5).

As shown in Figure 5, the segmentation results were more or less influenced by the existence of blood vessels with high gray level values that traversed through the boundaries of the nuclei in Nissl-stained sections. Figures 5G,H show the blood vessels at the border of the nucleus that then stretched to press the border toward the inside. Before a blood vessel had vanished, the border did not immediately retract, as shown in Figure 5E. When the blood vessel disappeared, the segmented boundary converged to the real boundary of the nucleus, as shown in Figure 5F. Note that our method was unable to fit the sharp hollows of the target's boundary very well due to the smoothness and continuousness of CCS in math, as shown in Figure 5E. Considering the different sizes of the aforementioned target nuclei, the computational parameters, namely, width (*w*), height (*h*) and sliding range (*r*), were set to different values (Table 2).

**Table 2:** **Computational parameters used to analyze the cytoarchitectural image data**.

	**Width (pixel)**	**Height (pixel)**	**Range (pixel)**
CENT2gr	100	200	60
CENT2mo	100	200	60
MOBmi	100	200	10
outline	50	25	10

### The outline segmentation of MRI/CT mouse brain images

**Figure 6:**
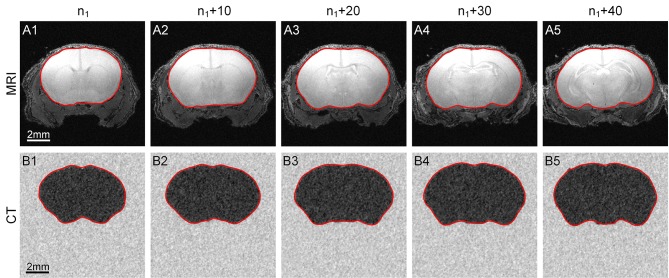
**Segmentation of MRI and CT data**. The results obtained by applying our method to MRI and CT data. **(A1–A5)** The 1st row shows the contour evolution process in MR images. **(B1–B5)** The 2nd row shows the process using CT data. The red curves are the computed CCS of the outline of the target area. n_1_ represents the 10th section of the image sequence of every dataset. The image to the right of each column is the 10th image after it.

Our method can be applied to MRI and CT data in addition to cytoarchitectural images. We used a C57BL/6 mouse brain MRI dataset (Bruker Biospect, 7.0 T/20 cm, 300 MHz) with a resolution of 60 μm/pixel and dimensions of 200 × 200 × 320. Fifty coronal sections were chosen for testing, and the results are shown in the first row of Figure 6.

We then selected a C57BL/6 mouse brain CT dataset (UltraBright, Oxford Instruments; PaxScan2520V, Varian Medical Systems. 50 kV, 40 W) with a resolution of 31.8 μm/pixel and dimensions of 800 × 800 × 800; 50 sections were chosen for our test. Note that the gray level range of the acquired original CT data was -0.402 to 0.136. We extended this range to 0–255 for convenience. The results are shown in the second row of Figure 6.

The parameters *w*, *h* and *r* used to analyze the MRI and CT data were set to 10, 5 and 3, respectively.

## Discussion

We developed a method to extract the boundaries of nuclei with relatively high cell density in an image sequence with CCS, which can also be used for the segmentation of nuclei with low cell density that are surrounded by areas with high cell density. Our method is also able to extract the boundaries of traditional non-nucleus objects.

Compared to our method, the classical segmentation algorithms used in the image segmentation field are difficult to apply to the segmentation of nuclei consisting of scattered cell bodies. The traditional thresholding method tends to extract the boundaries of isolated cells but not the whole boundary of the nucleus. The region-growing method is suitable for the segmentation of target areas with homogenous gray level distributions. The snake algorithm is easily disturbed by cells near the boundary, whereas the level set method tends to cause the boundary to converge onto isolated cells.

In the past, the boundaries of nuclei in high-resolution cytoarchitectural images were manually delineated based on experts' experience. With the emergence of high-resolution data-acquisition techniques, such as MOST, it has become unrealistic to draw lines on tens of thousands of sections by hand. Our method can automatically segment the boundaries of nuclei that differ in cell density to a high degree relative to their surrounding area, thereby partially automating the delineation of nucleus boundaries in high-resolution cytoarchitectural images and ensuring future application in the illustration of high-resolution brain atlases.

Our method has some limitations. In the mouse brain, many nuclei have complicated morphologies, and their boundaries occasionally split and merge along the coronal direction, which cannot be handled by our method. Our next goal is to extract nuclei with complicated morphologies and solve this problem by determining a series of conditions to detect the occurrence of the splitting and merging of nucleus boundaries.

## Author contributions

ZF and QL designed the proposed method, and ZF also coded the CSS toolkit and completed the performance testing. AL and HG provided the data needed for testing and giving many essential advices on articles writing and model testing. QL provided the support in hardware and given many essential advices. ZF drafted the manuscript and all authors reviewed and approved the final version of the manuscript.

### Conflict of interest statement

The authors declare that the research was conducted in the absence of any commercial or financial relationships that could be construed as a potential conflict of interest.
